# Defining Traceability Attributes and Consumers’ Segmentation Based on Fish Attributes: A Case Study in Italy and Spain

**DOI:** 10.3390/foods13162518

**Published:** 2024-08-12

**Authors:** Ahmed Saidi, Carla Cavallo, Sharon Puleo, Teresa Del Giudice, Gianni Cicia

**Affiliations:** Department of Agricultural Sciences, University of Naples Federico II, 80055 Portici, Italy; ahmed.saidi@unina.it (A.S.); sharon.puleo@unina.it (S.P.); teresa.delgiudice@unina.it (T.D.G.); cicia@unina.it (G.C.)

**Keywords:** segmentation, cluster analysis, consumer, behaviour, traceability, fish

## Abstract

Consumers’ involvement in the development of new goods and services is growing, and thus understanding food motives is crucial for various fisheries stakeholders to manage fish value chains. In addition, traceability is becoming more prominent in guiding consumers’ behaviour. Yet, the latter can be considered a source of confusion, as the multiplicity of certifications and labelling systems can be overwhelming. A national web-based survey was conducted on a representative sample of Italian and Spanish consumers. From the literature, a set of 13 attributes was chosen to identify the most important factors guiding consumers’ choice, and various groups in each population were identified using hierarchical cluster analysis. Our findings provide valuable insights for fish value chain stakeholders, enabling them to optimize fisheries supply chains, educate consumers about diverse fish species, and promote more sustainable decision-making.

## 1. Introduction

Consumers make food decisions every day [1]. In the recent decade, healthy eating habits have gained increasing attention [2,3,4,5]. Fish is recognized as an important component of a balanced and healthy diet, notably for its long-chain polyunsaturated fatty acids (LCPUFAs), such as eicosa-pentaenoic acid (EPA) and docosahexaenoic acid (DHA), that offer many benefits, including reducing the likelihood of cardiovascular diseases [6,7,8].

Recent studies have focused on understanding the impact of seafood attributes on consumer preferences and decision-making processes [9,10]. Yet, additional investigation is required to explore how various product attributes and consumer characteristics collectively influence consumer decision-making [9,10,11,12]. Evaluating these factors can be either positive or negative, representing drivers or barriers to fish consumption behaviour. Several determinants influence consumers’ attitudes towards fish, such as sensory characteristics, fish species, quality labels, sustainability concerns, price, catch area, and production method [13,14,15,16]. For example, positive drivers for fish consumption include factors such as good taste, freshness, ease of preparation, fish species, quality labels, catch area, and production method [11,16,17,18]. Conversely, significant barriers to fish consumption often originate from sensory aspects that consumers find undesirable, such as strong odours, disagreeable flavours or textures, and the inconvenience of bones. Price is also a significant barrier to seafood consumption, and seasonality and sustainability issues can also affect consumer attitudes towards fish [19,20,21,22].

In the last 20 years, fish producers and processors have mostly undertaken a product differentiation strategy to raise their competitiveness. With numerous health scares, consumers have become more aware of the potential health risks associated with consuming seafood that is not properly traced, as well as the negative environmental impacts that can result from unsustainable fishing practices [23]. In response, governments, industry organizations, and retailers are implementing traceability systems to ensure that seafood products are safe, sustainable, and accurately labelled [24,25]. These systems typically involve tracking the product from the point of catch or harvest all the way through to the final point of sale, using various technologies and data management tools [9,26,27] By providing more information about the origin, quality, and sustainability of seafood products, traceability systems can help build consumer trust and promote more responsible practices in the seafood industry [28].

In recent years, both Italy and Spain have seen a shift in consumer preferences for fish [29], with growing concerns about overfishing and sustainable and locally sourced products. This trend is driven by a number of factors, including concerns over overfishing, the environmental impact of fishing practices, and the quest for high-quality, fresh seafood [30,31]. In Italy, there has been a growing interest in traditional, regional fish dishes, particularly those made with lesser-known or underutilized species [28]. Consumers are also increasingly looking for fish that is certified sustainable and traceable, with a preference for products that are caught or farmed locally [32]. In Spain, there has been a similar trend towards sustainable and locally sourced fish [33], with an emphasis on high-quality products that are both fresh and affordable. 

Given the many options available to producers and processors to differentiate fish, we extend the current literature in two ways. First, we will assess the importance of traceability-related features when confronted with intrinsic and extrinsic cues. Then, we will provide insights regarding consumer segmentation in Italy and Spain to provide updated insights to policy-makers, marketers, and fish producers about consumer typologies.

The primary objective of this research is to explore the impact of traceability-related features on consumer preferences and purchasing behaviour. Specifically, we aim to investigate the relative importance of traceability-related features when compared to other intrinsic and extrinsic cues, such as price, quality, and origin.

Moreover, this study seeks to identify different consumer segments in Italy and Spain based on their attitudes and behaviours towards fish. By doing so, we aim to provide a more nuanced understanding of the various factors that drive consumer preferences and decision-making processes, which can inform targeted marketing strategies and policy interventions in the fish industry.

In this present study, the Best-Worst Scaling method (BWS) is used to evaluate key attributes defining consumer preferences for fish features [34]. This method provides a structured and systematic approach to measuring consumer preferences and can be used to identify distinct consumer segments based on their ranking of products and sociodemographic characteristics. 

The remainder of the paper is organized as follows: Section 2 provides an overview of the data collection and analysis. In Section 3, the results and discussions arising from the data analysis are reported, while in Section 4, the main conclusions are drawn.

## 2. Materials and Methods

### 2.1. Data Collection and Description 

Data were collected in 2021–2022 from online surveys and administered by two professional marketing agencies for a representative sample of Italian (*N* = 1003), and Spanish (*N* = 1000) respondents responsible for food shopping stratified by age, gender, population density and area of residence. The data collection method complied with EU and national ethical requirements as all respondents provided their informed consent to participate in the study, and all data were collected anonymously. The data were recorded and managed according to the “Italian Personal Data Protection Code” [35] and to the general data protection regulation of the European Union (Regulation (EU) 2016/679) (‘GDPR’) [36].

The questionnaire consisted of two parts. The first part collected respondents’ choice associated with the BW experiment, aimed at identifying consumer preferences for 13 finfish attributes selected from a recent systematic literature review [9] and exploratory research conducted in 4 Mediterranean countries [33] (Table A1, Appendix A). Fish species affect sustainability concerns, freshness, taste, cooking methods, and cultural significance, while the catch area signals freshness, quality, and ecological impact [37,38]. Seasonality enhances perceived quality and reduces environmental impact [39]. Wild-caught fish is often preferred for its natural quality, whereas farmed fish address sustainability concerns vs. freshness is a critical quality indicator, and sensory attributes like smell and appearance influence acceptability [40,41]. Convenience attributes such as being cleaned or filleted cater to ease of preparation [42]. Taste and consistency drive consumer satisfaction, and price affects purchasing decisions across segments [43,44]. The physical state of the fish impacts convenience and storage, while sustainable fishing practices address environmental and long-term availability concerns [45].

The second part of the questionnaire collected socio-demographic data of respondents (e.g., gender, age, education, number of individuals within the household, children under 12 years in the household self-declared income and diet), and fish consumption habits (the overall linking and the consumption frequency of fish in general. A nine-point Likert scale was used to assess consumers overall liking of fish, ranging from 1 = not at all to 9 = a lot. Fish consumption frequency was also assessed using a single-choice question with the following options: “Never”; “Once a year”; “Less than once a month”; “Once a month”; “2–3 times a month”; “Once a week”; “2 times a week”; and “More than 2 times a week”.

### 2.2. Best-Worst Method and Clustering Analysis

The Best–Worst Scaling (BWS) approach was used to classify consumers’ preferences for 13 fish traits. The BWS—also known as maximum difference scaling, was applied for the first time by Finn and Louviere [46] in a study on food safety. It consists of iteratively asking interviewees to choose the most preferred (“best”) and least preferred (“worst”) items of a choice set [47]. By requiring respondents to prioritize items, BW scaling solves the problem of many attributes having similar importance and gives clearer information about the ranking of choices [48]. The number of items in a choice set and the number of sets depend on the total number of items and the experimental design. Due to its unbiased nature, BWS has been widely used in studies on consumer behaviour [49,50], food preferences [51], wine marketing [34,52,53], and ethical beliefs [49]. Unlike other rating scales, BWS is free from cultural bias, making it a reliable and precise method for ranking consumer preferences [34].

The current BWS experiment had a balanced incomplete block design (13,4,4,1) (The choice set consists of 13 items, 4 repetitions per level, 4 items in each choice set, and 1 pair frequency), i.e., 13 items divided into 13 choice sets with four items each, and every attribute appearing 4 times in the choice sets. Balanced indicates that every item appears the same number of times. The 13 items fish preferences-related attributes are detailed in Table 1. Respondents were asked to choose between fish attributes according to which they considered the most (and least) important in their choice of fish (Table 1). 

The ranking of fish traits was computed for every respondent individually and ultimately for the full sample by allocating a + 1 to each attribute that was described as the best and a − 1 to each attribute that was mentioned as the worst. Adding the + 1 s and −1 s yielded a score (BW score) for each fish attribute, which was used to determine the final ranking. The experimental design was conducted in such a way that each subject received a score ranging from −4 to + 4 for each attribute. While the BW score shows the prominence of an attribute, negative values imply below-average preference rather than dislike [54].

A two-step cluster analysis was used to further analyze the heterogeneity underlying attribute importance among respondents and unveil patterns that may be used for market analysis [55,56]. First, Hierarchical cluster analysis was used to group the participants based on their demographic characteristics and preferences. Hierarchical cluster analysis presupposes that individuals belong to one of k clusters, the size and number of which are unknown a priori. Furthermore, hierarchical clustering implies that there are distinct clusters of consumers with similar preferences within segments but considerably different preferences across clusters [57,58]. Second, we used Ward’s linkage method to minimize the variance within each cluster and ensure that the resulting groups were as homogenous as possible. We identified homogeneous consumer groups in Italy and Spain using the Duda-Hart Je(2)/Je index to select the optimal number of segments [59]. ANOVA tests were undertaken to assess whether segments differed significantly in the importance of each attribute, using the BW score as an indicator of attribute-related importance. Specifically, ANOVA F statistics tests BW scores across clusters against the null hypothesis that they are statistically equal across clusters. Subsequently, post hoc Tukey tests investigated the pairwise statistically significant differences (*p* < 0.05) among the cluster means.

All analyses were performed using Stata 14.0 software.

## 3. Results and Discussion

### 3.1. Sample Description

In Italy, 506 females (50.4%) and 497 males (49.6%) completed the survey. Among the respondents, 298 were between 55 and 77 years old (29.7%), 285 were between 30 and 40 years old (28.4%), 241 were between 45 and 54 (24%), and 178 were between 18 and 29 years old (17.8%). A total of 532 respondents held high school diplomas (53%), 298 had bachelor’s degrees (29.7%), 106 were secondary school diploma holders (10.6%), and 32 and 35 had master’s and PhD degrees, respectively (3.2% and 3.5%). In terms of employment, 400 of the respondents were employees (39.9%), 198 were unemployed (19.7%), 113 were freelancers (11.3%), 76 were students (7.6%), and 99 had other professions (9.9%). Regarding their living areas, 515 respondents lived in internal areas (internal areas are typically rural areas that are not along the coast or near the mountains. They often lack access to essential services and infrastructure compared to urban or more developed regions [60]) (51.4%), 214 lived in seaside cities (seaside cities are urban areas located directly along the coast. They typically have a strong connection to maritime activities, including tourism, fishing, and trade [61]) (21.4%), 211 lived near the seaside (near the seaside, cities are situated close to but not directly on the coast. They benefit from proximity to the sea and its associated economic and recreational opportunities without being in the immediate coastal zone [60]) (21.1%), and 60 lived in mountainous areas (mountain areas are regions with a significant elevation and rugged terrain. These areas are crucial for biodiversity, water resources, and cultural heritage. They cover about 27% of the earth’s land surface and are home to diverse ecosystems and communities [62]) (6%). Most respondents, 784, did not have children (78.2%). The dietary preferences included 832 omnivores (82.9%), 62 pescatarians (6.2%), 55 flexitarians (5.5%), 21 vegetarians (2.1%), 7 vegans (0.7%), and 27 with diets related to personal food allergies and preferences (2.7%) (Table A2, Appendix A). 

In Spain, 503 males (50.3%) and 497 females (49.7%) completed the survey. Among the respondents, 350 were between the ages of 55 and 77 (35%), 261 were between 30 and 40 years old (26.1%), 150 were between 45 and 54 years old (15%), and 239 were between the ages of 18 and 29 (23.9%). A total of 394 respondents had a high school diploma (39.4%), 378 had bachelor’s degrees (37.8%), 110 had secondary school diplomas (11%), and 118 had Master’s or PhD degrees (11.8%). Regarding employment, 395 respondents were employees (39.5%), 207 were unemployed (20.7%), 84 were freelancers (8.4%), 97 were students (9.7%), and 108 worked in other fields (10.8%). In terms of living area, 407 respondents lived in internal areas (40.7%), 322 lived in seaside cities (32.2%), 227 lived near the sea (22.7%), and 44 lived in mountainous areas (4.4%). Additionally, 330 respondents did not have children (33%). Most respondents, 542, were omnivores (54.2%). (Table A2, Appendix A).

### 3.2. Average Best–Worst Score Analysis

The BW score was calculated by subtracting the number of times each attribute was rated as worst (W), least important, from the number of times it was rated as best (B), most important. This score was then divided by the total number of respondents (n) in the sample to determine the average BW score, represented as (B–W)/n.

Table 2 and Table 3 provide a summary of the best-to-worst scores for Italian and Spanish consumers. Italian consumers selected freshness, smell/appearance and taste/consistency as the most important attributes when making their decision. Italians rely on intrinsic product properties when making their choices. Generally, freshness is a key determinant in influencing fish consumption [21,63]. Consumers associate fresh fish with fewer health risks and minimal use of hormones and drugs during the production process. We found that Italian consumers rely on their own judgement rather than trust information provided by sellers (origin, production method, seasonality, price, fish species). Then, the attribute of sustainable fishing scored lower, suggesting that it is of secondary interest.

Conversely, Spanish consumers selected fish species, followed by farmed fish and cleaned/filleted traits, as the most important attributes when choosing fish. The exposure of Spanish consumers to fish might be the reason behind their huge interest in fish, its production method, and its commodity of use [64]. Spanish respondents placed a high value on fish that was ready to be cooked. In addition, farmed fish is usually more available compared to wild-caught alternatives, as it is accessible all year round and does not depend on seasonality [65]. Consumers may have an attitude–behaviour gap and choose the most convenient option over the fresh one [19,66]. Furthermore, price ranked fourth after the cleaned/filleted attribute. Although price is usually important, in this case the respondents were willing to pay more when more benefits were perceived, as quicker preparation, fitting the demands of modern lifestyles.

Globally, we can see that Italian and Spanish consumers have diametrically opposed preferences. While Italians ranked top in terms of sensory product qualities and freshness, Spanish consumers ranked lowest. Likewise, Spanish people scored higher for fish species, farmed fish, and cleaned/filleted features compared to Italians. Results yielded a lower importance for intrinsic characteristics by Spanish consumers that may be attributable to their higher familiarity with the product [67].

### 3.3. Cluster Analysis 

In Italy, four groups of consumers were defined as: “Coastal consumers”, “Traceability enthusiasts”, “Sensory sensitive consumers”, and “Convenience enthusiasts” (Table 4 and Table 5).

The first group of Italian consumers were dubbed “*Coastal consumers*” (29% of the sample). The most significant positive attributes identified for this group were freshness (2.92), physical state (1.2), and being wild-caught (1.24). While seasonality was of lesser concern (−0.94). This cluster mainly lives in a seaside city or near the seaside (2.3). The positive perception of freshness, wild-caught, and physical state attributes suggests a preference for locally sourced seafood. However, their lower concern for seasonality (−0.94) may indicate a preference for consistent availability over seasonal variation. In line with previous studies by Murray et al. (2017) [20] and Temesi et al. (2020) [68], exposure, particularly during childhood, influences consumers to continue eating fish throughout their lives and makes them more discerning about the attributes they personally value. The main pattern characterizing fish consumption is linked to proximity to the seaside, as people living near the sea generally have a higher fish consumption compared to inland residents [33,69,70]. *Coastal consumers* considered price non-significant when buying fish; the high income of individuals in this group made them less sensitive to the expensive nature of fish. Although previous studies demonstrated the importance of price when buying fish [22], dietary habits appear to exert greater influence than price when consumers make decisions about purchasing fish [20,71]. As the most significant consumers of fish among the identified clusters, seaside residents have a unique opportunity to embrace diversified fish-eating practices. Marketers can leverage this proximity to the ocean by promoting a variety of locally sourced fish and seafood options. This not only supports sustainable fishing practices but also encourages a richer and more diverse diet. By highlighting the freshness, health benefits, and culinary versatility of different species, marketers can inspire seaside communities to explore new and exciting ways to enjoy their abundant marine resources”.

The second Italian consumer group, “*Traceability enthusiasts*” (13% of the sample), valued quality label (1.93), and sustainable fishing (2.26) the most, while they were less interested in whether fish was cleaned/filleted (−2.21), whether fish is farmed (−1.01), and price (−2.11). In addition, consumers in this group had the highest average for fish preference (8.3) and fish frequency (5.4) consumption compared to all other segments. Traceability enthusiasts regrouped people who were older people (2.8), with large families (3.0), and living near the seaside (2.5). A clear connection between price and ecolabelling does exist, in line with previous studies where consumers were willing to pay more for an eco-labelled or socially responsible product [27,72]. In addition, this group’s interest in fishing can be attributed to a demand for a quality label that generally guarantees ethical exploitation of marine resources and sustainable fishing practices [73]. Furthermore, in line with previous studies, consumers’ interest in fish attributes is strongly driven by biological and socio-demographic factors, with older individuals being most likely interested in fish labels and product safety due to higher health concerns and sustainability claims [74,75]. 

The third group of Italian consumers, “*Sensory-sensitive consumers*” (35% of the sample) was the largest. Sensory-sensitive consumers show the highest appreciation for smell/appearance (1.91), taste/consistency (1.55), and price (0.88). These attributes play a crucial role in their decision-making process, highlighting a preference for sensory appeal and affordability. In contrast, they place less emphasis on factors such as sustainable fishing practices (−0.54), wild caught status (−0.12), seasonality (−0.55), farmed fish (−0.75), catch area (−0.24), and specific fish species (−1.40). This suggests that while sensory qualities and cost are significant influencers for this group, consumers in this cluster are less motivated by environmental concerns when making purchasing decisions [76,77]. This could be due to a lack of trust or knowledge about certain catch areas or a general indifference to the geographical origin of the products. The low educational level in this group may also contribute to the lack of interest in sustainability-related attributes. Previous studies by Can et al. (2015) [78], Uddin et al. (2020) [79], and Myrland et al. (2000) [80]. Thus, educating consumers by promoting underutilized fish species throughout mass and social media could help to revive the consumption of forgotten or cheaper fish species currently neglected due to health claims around popular fish species [14,81].

Lastly, the fourth group of Italian consumers, “*Convenience enthusiasts*” (23% of the sample), place a strong emphasis on convenience, as indicated by their positive preference for cleaned and filleted fish (0.16). They prioritize convenience over freshness (1.15), which is of lesser concern to them compared to other clusters. They also show a low concern for farmed fish (−0.75), and they exhibit less interest in fish species (−0.55) and catch area (−0.24) compared to the other attributes, suggesting that while they value convenience, they are also somewhat discerning about the types and origins of the fish they consume. Additionally, they had the lowest preference (6.4) and consumption frequency (4.5) of fish among all clusters, leading to their lower seafood product requisitions. *Convenience enthusiasts* were the youngest and had the lowest income level compared to the other groups. Many scholars demonstrated a generational gap in consumers’ food preferences, as younger people were found to be more open in their fish consumption habits [19,82,83]. Furthermore, higher income levels were generally associated with higher dietary fish intake [84]. 

In Spain, four groups of consumers were defined as: “Value-Conscious Consumers,” “Affluent Convenience Enthusiasts,” “Quality-Indifferent Consumers” and “Sustainability-Conscious consumers” (Table 6 and Table 7).

The first group of Spanish consumers were “*Value-Conscious Consumers*” (27% of the sample). These consumers highly value the price (1.86) and the convenience of cleaned/filleted fish (1.80). Physical state (0.50) and seasonality (0.01) are also important, though to a lesser extent. Conversely, they place less emphasis on quality label (−1.54), taste/consistency (−0.60), smell/consistency (−0.56), sustainable fishing (−0.43), and catch area (−0.18). This group had the highest preference for and consumption of fish compared to other groups, suggesting that they prioritize affordability and convenience over quality indicators and sustainability factors, in line with findings from Cantillo et al. (2020) [10], Saidi et al. (2022) [9], and Onyeneke et al. (2020) [85]. To effectively engage *Value-Conscious Consumers*, marketers should emphasize affordability and convenience, highlight competitive pricing, offer promotional deals, and display pre-cleaned and filleted fish products. Ensuring products are visually appealing and well-maintained will also cater to their preference for physical state. While seasonality is less important, introducing seasonal varieties can add novelty [86].

The second group of Spanish consumers, termed the “*Affluent Convenience Enthusiasts*” (24% of the sample), positively valued catch area (0.11) and cleaned/filleted fish (1.05), indicating a preference for convenience and knowing the origin of their fish. They valued less freshness (−2.74), seasonality (−0.39), taste/consistency (−1.78), and smell/appearance (−1.95). Additionally, this cluster showed the second-highest preference (8.00) and consumption (5.97) of fish out of the four clusters and had the highest income levels compared to the other identified clusters. In line with previous studies highlighting the importance of income level for consumers fish choice [78,79,84], the effect of price, found to be significant for this cluster, seems to overlap with the level of income, suggesting that *Affluent Convenience Enthusiasts* prioritize practical aspects and convenience over sensory qualities and freshness, potentially seeking consistent and easily prepared options rather than high-quality or seasonal products. To effectively engage “Affluent Convenience Enthusiasts,” marketers should emphasize the convenience of cleaned and filleted fish and promote the traceability of the catch area. Offering premium, ready-to-cook meal options can cater to their high income levels and preference for ease. Additionally, educational content on the benefits of freshness and quality can gradually shift their perceptions towards healthier and more conscious fish choices rather than focusing on consistent and easily prepared options. 

The third group of Spanish consumers, dubbed “*Quality-Indifferent Consumers*” (23% of the sample), value less cleanliness and filleting of fish (−0.43), quality labels (−0.65), catch area (−0.13), and freshness (−1.09). This group preferred whole fish compared to filleted fish products. They also had the fewest children in their households (1.55), indicating their practical and potentially adult-focused meal preferences. In addition, this group lived mainly in inland and mountainous areas where fish supply is more challenging compared to seaside areas [87]. *Quality-Indifferent Consumers* preferred (6.15) and consumed (4.65) fish the least compared to the other clusters. Similar to Cantillo et al. (2020) [10], Smith et al. (2017) [88], and Liu et al. (2015) [89], those with more kids are more careful about increasing fish consumption and its quality and safety. Large families also often prioritize budget-friendly food options [85,90,91]. Therefore, focusing on the value-for-money aspect of fish products while ensuring they meet taste and quality expectations can encourage *Quality-Indifferent Consumers* to increase their fish consumption. Since cleaned and filleted fish are less appealing to this group, offering whole fish or minimally processed options at lower prices may better align with their preferences and make fish more attractive. Improving the availability and distribution of affordable fish products in inland and mountainous areas is crucial, as residents far from the coast often encounter challenges accessing seafood [33].

The fourth and last group of Spanish consumers were dubbed “*Sustainability-Conscious Consumers*” (26% of the sample). They valued fish species (1.73) the highest, followed by quality labels (0.74), cleaned/filleted fish (0.45), physical state (0.25), and catch area (0.17), indicating a preference for knowing the origin of their fish. They also valued sustainable fishing (0.08). However, they placed a significant negative value on price (−2.27), suggesting a lower focus on affordability when making their decision. Sensory attributes such as taste/consistency (−1.39) and smell/appearance (−1.31) were less valued by this group. The lower income levels (2.03), smaller family sizes (2.86), and higher number of children (1.78), further underscore their focus on sustainable eating choices. This behaviour reflects a practical approach that emphasizes sustainability and essential qualities over cost and sensory attributes, aligning with their family-oriented consumption patterns and financial constraints, in line with previous findings by Bronnmann et al. (2018) [92] and Onyeneke et al. (2020) [85]. To better approach this group, marketers should highlight fish species and quality labels through clear labelling and educational campaigns, emphasizing catch areas and sustainable practices. Offering family-oriented promotions like value packs and cooking workshops can cater to their practical needs. Building trust through storytelling and customer testimonials, and engaging them with tasting events and interactive content, will create a more personal connection. 

In Italy, all four clusters considered freshness a fundamental element in their purchasing decisions. This outcome could be due to an overlap between sensory attributes, physical features, and freshness, as previous scholars have found out the importance of fresh fish and its association with several intrinsic and extrinsic traits like health aspects, taste, quality, and origin in shaping consumers’ preferences [9,70,93,94]. “Convenience enthusiasts,” on the other hand, were less interested in the freshness of fish. This could be due to the typology of consumers in this segment, who are younger and considered healthier and therefore less concerned about what they eat or do not perceive any important attribute in that innovative product [71,95]. While Spanish consumers did attach importance to the freshness and sensory characteristics of fish, their decision-making depended more on the type of fish, the production method, and convenience. The choice of Spanish consumers could be related to the availability of seafood in Spain, as Spain is one of the most important European fishing countries in terms of production, employment, fleet, consumption, and aquaculture [96,97].

## 4. Conclusions

In this study, we employed Best-Worst Scaling (BWS) analysis to identify the most significant fish attributes guiding Italian and Spanish consumers in their decision-making process. Additionally, we defined four clusters for each population based on traceability features, intrinsic and extrinsic cues, and sociodemographic factors.

The results highlight significant differences in the fish attribute preferences of Italian and Spanish consumers. Italian consumers prioritize sensory qualities and freshness, aligning with their culinary traditions that emphasize fresh and high-quality ingredients. In Italy, a strong emphasis on regional specialties like Sicilian anchovies and Adriatic bream reflects a preference for local, seasonal catches [98]. In contrast, Spanish consumers show a preference for attributes that emphasize convenience and availability, such as fish species, farmed fish, and cleaned/filleted options. These preferences reflect a practical approach to fish consumption and a greater familiarity with the product, as Spain’s diverse coastline supports a broad spectrum of seafood choices, from traditional dishes like bacalao to popular species such as hake and tuna [99,100,101]. The divergence in preferences between both countries suggests that marketing strategies and product offerings should be tailored to each market, with Italians responding more to freshness and quality, while Spaniards favour convenience and availability. Understanding these distinct preferences is crucial for stakeholders in the fish industry to effectively cater to the needs of different markets, ultimately enhancing consumer satisfaction and driving sales.

The results of the cluster analysis reveal distinct and fragmented consumer preferences for fish, largely influenced by sociodemographic characteristics. Age, income, education level, place of residence, and household size determine both consumers’ responses to the different fish attributes and consumption levels. The findings suggest that targeted marketing strategies and tailored product offerings based on demographic factors may be more effective in promoting seafood consumption among Italian and Spanish consumers. Retailers and producers can use this information to better understand and meet the needs and preferences of different consumer groups, potentially increasing sales and improving consumer satisfaction. 

While traceability is important, it primarily concerns a smaller, more affluent segment of Italian and Spanish consumers. Implementing traceability technologies is time and effort-intensive [102], which may not be cost-effective for a limited consumer base. Instead, focusing on high-value fish products, such as Alaskan king crab, bluefin tuna, and beluga caviar, can optimize returns on investments while meeting the quality and transparency expectations of discerning consumers.

Our findings are not free from limitations. One of these shortcomings is related to the way the attributes used for the BW experiment were described and how individual consumers interpreted them. In addition, the BW method uses an unrealistic map experiment, which could reduce the external validity of the results. Furthermore, the order in which items are presented in a BWS survey can influence the responses. Consumers may be more likely to choose the “best” item if it is presented first or the “worst” item if it is presented last. Moreover, consumers’ choices in a BWS survey may be influenced by the context in which the survey is conducted, such as the time of day or the consumer’s mood. Lastly, BWS results may not be generalizable to other contexts or populations, as preferences and priorities can vary across different groups of consumers. In an attempt to overcome one of the main limitations of the current study, it would be worthwhile to analyze consumers’ attitudes and preferences for selected fish attributes defined in more detail. A more realistic research design for higher external validity of the results is needed. This could be achieved, for example, through the use of experimental markets or grocery stores to replicate the features of a real-world marketplace, such as the physical layout, product assortment, and pricing structures [103]. Combining BWS with other research methods, such as focus groups or in-store experiments, in future studies to gain a more comprehensive understanding of consumer preferences or exploring alternative methods for measuring consumer preferences, such as conjoint analysis or adaptive choice-based conjoint analysis, might offer greater scale sensitivity and reduce order bias. Finally, the outcomes of the present study may not be transferable to other geographical contexts. Thus, conducting BWS surveys in multiple contexts or with diverse populations will contribute to assessing the generalizability of the results, particularly within the Mediterranean region. 

## Figures and Tables

**Table 1:** Example of a filled-out choice set.

	Most Important	Least Important
Fish species	x	
Price		
Physical state (fresh, frozen, defrosted)		
Sustainable fishing		x

**Table 2:** Italian Sample-level BW scores and average BW scores.

Fish Attributes	Best Scores	Worst Scores	BW Scores	Average BW Scores	Sqrt |B/W|	Standardized Ratio Scale (%)	Standardized Importance Weights Scale (%)
Freshness	2527	−166	2360	2.35	3.90	100	22
Smell/appearance	1708	−279	1428	1.42	2.47	64	14
Taste/consistency	1354	−278	1075	1.07	2.21	57	12
Quality label	892	−206	686	0.68	2.08	53	12
wildly caught fish	1080	−596	485	0.48	1.35	35	8
Sustainable fishing	1016	−737	280	0.27	1.17	30	7
Physical state	1350	−1024	326	0.32	1.15	29	6
Seasonality	783	−1208	−424	−0.42	0.81	21	5
Price	668	−1328	−659	−0.65	0.71	18	4
Cleaned/filleted	565	−1749	−1185	−1.18	0.57	15	3
Catch area	481	−1724	−1243	−1.23	0.53	14	3
Fish species	341	−1465	−1124	−1.12	0.48	12	3
Farmed fish	202	−1437	−1236	−1.23	0.37	10	2
Total					17.80		100

**Table 3:** Spanish sample-level BW scores and average BW scores.

Fish Attributes	Best Scores	Worst Scores	BW Scores	Average BW Scores	Sqrt B/W	Standardized Ratio Scale	Standardized Importance Weights Scale
Fish species	1541	−207	1334	13.34	2.73	100	19
Farmed fish	1067	−172	895	8.95	2.49	91	17
Cleaned/filleted	1456	−707	749	7.49	1.44	53	10
Price	1276	−863	413	4.13	1.22	45	8
wildly caught fish	915	−763	152	1.52	1.10	40	7
Seasonality	907	−815	92	0.92	1.05	39	7
Catch area	423	−431	−8	−0.08	0.99	36	7
Physical state	893	−999	−106	−1.06	0.95	35	6
Quality label	816	−1037	−221	−2.21	0.89	32	6
Sustainable fishing	115	−232	−117	−1.17	0.70	26	5
Smell/appearance	332	−1523	−1191	−11.91	0.47	17	3
Taste/consistency	291	−1436	−1145	−11.45	0.45	16	3
Freshness	157	−2281	−2124	−21.24	0.26	10	2
Total					14.73	539	100

**Table 4:** Heterogeneity of preferences for product attributes according to BW scores, Italy (The asterisk (*) in the last column indicates a F test *p*-value < 0.05, rejecting the null hypothesis of equality of mean values across groups. BW scores bearing the same letter on the same row were not significantly different according to pairwise Tukey test (*p* < 0.05)).

Clusters	Coastal Consumers (*N* = 287)	Traceability Enthusiasts (*N* = 136)	Sensory-Sensitive Consumers (*N* = 351)	Convenience Enthusiasts (*N* = 229)	F Stats
Quality label	0.62 (b)	1.93 (a)	0.28 (c)	0.64 (b)	62.90 *
Fish species	−1.23 (b)	−1.13 (b)	−1.40 (b)	−0.55 (a)	15.44 *
Catch area	−1.39 (c)	−0.76 (b)	−1.96 (d)	−0.24 (a)	59.09 *
Freshness	2.92 (a)	2.07 (c)	2.78 (b)	1.15 (d)	100.47
Price	−1.97 (c)	−2.11 (c)	0.88 (a)	−0.51 (b)	187.30 *
Physical state	1.24 (a)	−0.80 (d)	0.49 (b)	−0.41 (c)	55.46 *
Sustainable fishing	0.48 (b)	2.26 (a)	−0.54 (d)	0.11 (c)	94.32 *
Wildly caught fish	1.20 (a)	0.84 (b)	−0.12 (d)	0.31 (c)	38.85 *
Seasonality	−0.94 (c)	0.70 (a)	−0.36 (b)	−0.55 (b)	28.89 *
Farmed fish	−1.01 (b)	−1.40 (c)	−1.67 (d)	−0.75 (a)	32.88 *
Cleaned/filleted	−2.25 (c)	−2.21 (c)	−0.79 (b)	0.16 (a)	99.37 *
Taste/consistency	1.24 (b)	0.51 (c)	1.55 (a)	0.46 (c)	43.46 *
Smell/appearance	1.83 (a)	0.28 (c)	1.91 (a)	0.85 (b)	59.06 *

**Table 5:** Cluster differences in terms of respondent socio-demographics and fish consumption habits, Italy (The asterisk (*) in the last column indicates a F test *p*-value < 0.05, rejecting the null hypothesis of equality of mean values across groups. BW scores bearing the same letter on the same row were not significantly different according to pairwise Tukey test (*p* < 0.05)).

Clusters	Coastal Consumers (*N* = 287)	Traceability Enthusiasts (*N* = 136)	Sensory-Sensitive Consumers (*N* = 351)	Convenience Enthusiasts (*N* = 229)	F Stats
Fish preference	8.3 (a)	8.1 (a)	7.7 (b)	6.4 (c)	63.25 *
Fish frequency consumption	5.4 (a)	5.4 (a)	5.0 (b)	4.5 (c)	25.79 *
Sex	1.5 (a)	1.4 (a)	1.5 (b)	1.6 (a)	1.80
Age range	2.8 (a)	2.8 (a)	2.6 (b)	2.4 (c)	9.52 *
Living area	2.3 (c)	2.5 (a)	2.5 (a,b)	2.4 (a)	4.30 *
Education	2.3 (a)	2.5 (a)	2.3 (a)	2.4 (a)	1.99
Job	3.3 (a)	2.9 (b,c)	3.1 (b)	2.6 (c)	10.44 *
Income	2.8 (a)	2.4 (b)	2.5 (b)	2.4 (b)	4.05 *
Family	3.2 (a)	3.0 (a)	2.9 (b)	3.1 (a)	4.22 *
Kids	1.8 (a)	1.8 (a)	1.8 (a)	1.7 (a)	4.95 *
Diet	3.3 (a)	3.2 (a)	3.2 (b)	3.2 (a)	2.01

**Table 6:** Heterogeneity of preferences for product attributes according to BW scores, Spain (The asterisk (*) in the last column indicates a F test *p*-value < 0.05, rejecting the null hypothesis of equality of mean values across groups. BW scores bearing the same letter on the same row were not significantly different according to pairwise Tukey test (*p* < 0.05)).

Clusters	Value-Conscious Consumers (*N* = 268)	Affluent Convenience Enthusiasts (*N* = 240)	Quality-Indifferent Consumers (*N* = 234)	Sustainability-Conscious Consumers (*N* = 258)	F Stats
Quality label	−1.54 (c)	0.63 (a)	−0.65 (b)	0.74 (a)	100.06 *
Fish species	1.00 (b)	1.60 (a)	1.00 (b)	1.73 (a)	18.21 *
Catch area	−0.18 (d)	0.11 (b)	−0.13 (c)	0.17 (a)	8.46 *
Freshness	−2.29 (b)	−2.74 (c)	−1.09 (a)	−2.32 (b)	68.14 *
Price	1.86 (a)	1.17 (b)	0.94 (b)	−2.27 (c)	300.31 *
Physical state	0.50 (a)	−0.72 (c)	−0.57 (c)	0.25 (b)	35.84 *
Sustainable fishing	−0.43 (c)	−0.09 (b)	0.00 (b)	0.08 (a)	45.37 *
Wildly caught fish	−0.71 (b)	0.48 (a)	0.66 (a)	0.27 (a)	40.83 *
Seasonality	0.01 (b)	−0.39 (c)	0.38 (a)	0.36 (a)	14.89 *
Farmed fish	0.97 (a)	1.10 (a)	0.62 (b)	0.88 (b)	8.30 *
Cleaned/filleted	1.80 (a)	1.05 (b)	−0.43 (d)	0.45 (c)	69.21 *
Taste/consistency	−0.60 (a)	−1.78 (d)	−0.84 (b)	−1.39 (c)	39.68 *
Smell/appearance	−0.56 (a)	−1.95 (d)	−1.00 (b)	−1.31 (c)	35.18 *

**Table 7:** Cluster differences in terms of respondent socio-demographics and fish consumption habits, Spain. (The asterisk (*) in the last column indicates a F test *p*-value < 0.05, rejecting the null hypothesis of equality of mean values across groups. BW scores bearing the same letter on the same row were not significantly different according to pairwise Tukey test (*p* < 0.05)).

Clusters	Value-Conscious Consumers (*N* = 268)	Affluent Convenience Enthusiasts (*N* = 240)	Quality-Indifferent Consumers (*N* = 234)	Sustainability-Conscious Consumers (*N* = 258)	F Stats
Fish preference	8.02 (a)	8.00 (a)	6.15 (b)	7.01 (c)	68.57 *
Fish frequency consumption	6.01 (a)	5.97 (a)	4.65 (c)	5.00 (b)	82.11 *
Sex	1.50 (a)	1.51 (a)	1.51 (a)	1.48 (a)	0.18
Age range	2.91 (a)	2.78 (a,b)	1.85 (c)	2.84 (b)	47.42 *
Living area	2.09 (b)	2.14 (b)	2.32 (a)	2.16 (b)	2.75 *
Education	2.47 (b)	2.73 (a)	2.39 (b)	2.43 (b)	8.41 *
Job	3.05 (b)	2.93 (b)	3.03 (b)	3.61 (a)	10.85 *
Income	2.14 (b)	2.83 (a)	2.10 (b)	2.03 (c)	18.70 *
Family	2.97 (b)	3.05 (b)	3.43 (a)	2.86 (c)	10.04 *
Kids	1.63 (c)	1.70 (b)	1.55 (d)	1.78 (a)	11.19 *
Diet	3.97 (a)	3.96 (a)	3.85 (a)	3.97 (a)	0.40

## Data Availability

The original contributions presented in the study are included in the article, further inquiries can be directed to the corresponding author.

## Appendix A

**Table A1:** Fish attributes used in the survey.

Attribute Categories	Fish Attribute	Description	References
Traceability related cues	Quality label (MSC, BAP, FOS, ASC)	The quality labels (MSC, BAP, FOS, ASC) are for products grown and processed product conditions.	[41,42,43]
	Fish species	Fish species are the various fish types available in the market	[44,45,46]
	Catch area	The place of origin where fish is caught (e.g., sea, river)	[13,43,47,48]
	Seasonality	Seasonal fish is caught only in the period that is dedicated for it.	[49]
	Wild caught fish	Fish caught in the sea.	[50]
	Farmed fish	Fish that originates from aquaculture.	[50,51]
Intrinsic cues	Freshness	Fish freshness is related to product intrinsic and extrinsic cues (e.g., freshly caught, good appearance, taste and quality)	[52,53,54,55]
	Smell, appearance	The smell of fish (e.g., not smelly, very smelly)	[56,57,58]
	Cleaned/ filleted	Fish cleaned and filleted	[59,63]
	Taste, consistency	The sensory features and consistency	[54,56,64]
Extrinsic cues	Price	The paid price	[45,46,65,66]
	Physical state	Physical state of fish (e.g., fresh, frozen, defrosted)	[67]
	Sustainable fishing	Sustainable fishing respects the laws against overexploitation.	[41,68,69]

**Table A2:** Summary statistics (N = 2003).

	Italy		Spain		Sig.
	N	%	N	%	
Gender					t = −0.3349, *p*-value (t > 0.05)
Male	506	50.4	497	49.7	
Female	497	49.6	503	50.3	
Age class					t = −0.6760, *p*-value (t > 0.05)
18–29	179	17.8	239	23.9	
30–44	285	28.4	261	26.1	
45–54	241	24	150	15	
55–70	298	29.7	350	35	
Educational level					t = 0.8847, *p*-value (t > 0.05)
Secondary school diploma	106	10.6	110	11	
High school diploma	532	53	394	39.4	
Bachelor’s degree	298	29.7	378	37.8	
Master or PhD	67	6.7	118	11.8	
Profession					t = −2.1306, *p*-value (t > 0.05)
Freelancer	113	11.3	84	8.4	
Employee	400	39.9	395	39.5	
Worker	117	11.7	107	10.7	
Unemployed	198	19.7	209	20.9	
Student	76	7.6	97	9.7	
Others	99	9.9	108	10.8	
Revenue level					t = 4.1918, *p*-value (t > 0.05)
<20.000€	271	27	311	31.1	
20.000–40.000€	408	40.7	413	41.3	
40.000–60.000€	126	12.6	135	13.5	
60.000–100.000€	41	4.1	52	5.2	
>100.000€	12	1.2	16	1.6	
Prefer to not respond	145	14.5	73	7.3	
Area of living					t = −1.1857, *p*-value (t > 0.05)
Seaside city	215	21.4	322	32.2	
Near the seaside	212	21.1	227	22.7	
Internal area	516	51.4	407	40.7	
Mounting area	60	6	44	4.4	
Kids					t = 5.6430, *p*-value (t > 0.05)
No	784	78.2	330	33	
Yes	219	21.8	670	67	
Number of household members					t = −1.1857, *p*-value (t > 0.05)
1	83	8.3	81	8.1	
2	266	26.5	269	26.9	
3	288	28.7	290	29	
4	305	30.4	269	26.9	
5	53	5.3	61	6.1	
6	6	0.6	19	1.9	
7	1	0.1	6	0.6	
8	1	0.1	2	0.2	
9	0	0	3	0.3	
Food orientation					t = −14.4102, *p*-value (t > 0.05)
Vegetarian	21	2.1	28	2.8	
Vegan	7	0.7	10	1	
Omnivore	831	82.9	542	54.2	
Flexitarian	55	5.5	110	11	
Pescatarian	62	6.2	36	3.6	
Others	27	2.7	274	27.4	
Total	1003	100	1000	100	
